# The presence of Quina lithic technology in China 50 to 60 ka ago remains a hypothesis

**DOI:** 10.1073/pnas.2518182122

**Published:** 2025-09-26

**Authors:** Laurence Bourguignon, Sylvain Soriano, Émilie Claud, Jean-Philippe Faivre, Eugénie Gauvrit-Roux, Jacques Jaubert, Liliane Meignen, Erwan Vaissié, Guillaume Guérin

**Affiliations:** ^a^Institut National de Recherches Archéologiques Préventives, Centre for archaeological research, Villeneuve-les-Béziers 34500, France; ^b^CNRS, Archéologies et Sciences de l’Antiquité, Anthropologie des Techniques, des Espaces et des Territoires MSH: Maison des Sciences de l’Homme, UMR 7041, MSH mondes, Université Paris Nanterre 92000, France; ^c^Institut National de Recherches Archéologiques Préventives, Centre for archaeological research, Clermont-Ferrand 63000, France; ^d^CNRS, Université de Bordeaux, De la Préhistoire à l’Actuel: Culture, Environnement et Anthropologie, UMR 5199, Pessac 33600, France; ^e^CNRS, Université de Rennes, Centre de Recherche en Archéologie, Archéosciences, Histoire, UMR 6566, Rennes 35000, France; ^f^CNRS, Université Côte d’Azur, Cultures – Environnements. Préhistoire, Antiquité, Moyen Âge, UMR 7264, Nice 06000, France; ^g^PaleoTime, Villard-de-Lans 38250, France; ^h^CNRS, Université de Rennes, Géosciences Rennes, UMR 6118, Rennes 35000, France

A recent article (1) suggests that the presence of thick scrapers with scalariform retouch at Longtan (China) reflects a European technical influence, possibly even migratory flows associated with Quina Mousterian. In our view, this hypothesis rests on fragile methodological grounds: partial morpho-typological reading, lack of in-depth technological analysis, uncertain chronological framework, and insufficiently addressed taphonomic issues.

First, morphological similarity alone is insufficient to establish cultural affiliation. Tools with scalariform retouch and/or significant thickness can arise independently without implying that the groups shared identical traditions or transmitted specific know-hows. Such similarities can reflect *technical convergence*, not cultural inheritance [e.g., the resemblance between Quina Mousterian and the Yabrudian from the Near East (2)]. Only a detailed operational sequence (*chaîne opératoire*) analysis—integrating volumetric structuring, production logic, and functional objectives—can adequately address such questions.

Second, the absence of techno-economic demonstration seriously weakens the diffusion hypothesis. The European Quina Mousterian (Late Middle Palaeolithic) is characterized by a highly structured operational sequence, governed by specific, well-documented production rules (3–6). This system can be broadly compared to the *Système par Surface de Débitage Alterné*, both systems relying on a simple, recurring algorithm [referred to as “basic” (7) or of “type C” (8)]. However, Quina Mousterian differs through its targeted objectives (transversal or axial asymmetry), which impose a distinctive rhythm of double alternation in surface exploitation. The algorithm described for the Longtan material could be a variant of the Discoid flaking method (9)—a possibility the authors overlook.

Third, the proposed OSL ages (50 to 60 ka), which underpin the transcontinental cultural continuity hypothesis, rely on the selective retention of high-dose grains. While modern plowing affected the subsurface layer of the site, it is suggested that “such a process could have been continuously active since the deposition of the sediments,” thereby contaminating Pleistocene sediments with more recent grains. The exclusion of 40 to 80% of grains as statistical “outliers” based on this unproven, yet critical assumption of vertical contamination, results in ages of sediment deposition that are probably significantly overestimated.

Fourth, the artefacts’ spatial distribution across noncumulative units and the important surface alterations of the artifacts (unorganized striations, dispersed rounding) point to complex taphonomic mechanical processes—whose contribution to scars interpreted as resulting from tool use is little discussed.

Considering these impediments, proposing a direct connection with European Mousterian traditions around 60 ka ago appears methodologically unsound. While some typological similarities may seem suggestive, they are insufficient to justify affiliation with the Quina techno-complex. Such a claim demands rigorous demonstration—technological, functional, and chronological. In their absence, claiming the presence of Quina lithic technology in China 50 to 60 ka ago appears premature and potentially misleading.

Finally, such a globalizing interpretation risks projecting euro-centered frameworks onto East Asian assemblages, may obscure local cultural dynamics and promote unfounded narratives of cultural continuity—at the expense of a contextualized understanding that respects regional specificities. In our view, the material from Longtan is interesting enough in itself not to generate speculations on Late Pleistocene human dynamics in East Asia.
